# Depth variations of P-wave azimuthal anisotropy beneath Mainland China

**DOI:** 10.1038/srep29614

**Published:** 2016-07-19

**Authors:** Wei Wei, Dapeng Zhao, Jiandong Xu, Bengang Zhou, Yaolin Shi

**Affiliations:** 1 Key Laboratory of Active Tectonics and Volcano, Institute of Geology, China Earthquake Administration, Beijing 100029, China; 2 Department of Geophysics, Tohoku University, Sendai 980-8578, Japan; 3 Key Laboratory of Computational Geodynamics, Chinese Academy of Sciences, Beijing 100049, China

## Abstract

A high-resolution model of P-wave anisotropic tomography beneath Mainland China and surrounding regions is determined using a large number of arrival-time data recorded by the China seismic network, the International Seismological Centre (ISC) and temporary seismic arrays deployed on the Tibetan Plateau. Our results provide important new insights into the subducted Indian plate and mantle dynamics in East Asia. Our tomographic images show that the northern limit of the subducting Indian plate has reached the Jinsha River suture in eastern Tibet. A striking variation of P-wave azimuthal anisotropy is revealed in the Indian lithosphere: the fast velocity direction (FVD) is NE-SW beneath the Indian continent, whereas the FVD is arc parallel beneath the Himalaya and Tibetan Plateau, which may reflect re-orientation of minerals due to lithospheric extension, in response to the India-Eurasia collision. There are multiple anisotropic layers with variable FVDs in some parts of the Tibetan Plateau, which may be the cause of the dominant null splitting measurements in these regions. A circular pattern of FVDs is revealed around the Philippine Sea slab beneath SE China, which reflects asthenospheric strain caused by toroidal mantle flow around the edge of the subducting slab.

Mainland China and adjacent regions are surrounded by convergent zones of the Indian, Eurasian, Pacific, Philippine Sea and North American plates (Fig. 1), which are characterized by intense ground deformations^1^,^2^ and widespread seismic and volcanic activities. The tectonics of western China has been controlled by the collision and continuous convergence of India and Eurasia since the Cenozoic^3^,^4^. During this process, at least 1400 km and perhaps as much as 2500 km of north-south shortening has been absorbed by crustal thickening of the Himalaya-Tibet orogen^4^ and the lateral extrusion of tectonic blocks along great strike-slip faults^5^. Global positioning system (GPS) measurements show that at present the dominant deformation of the plateau interior is an overall ESE-WNW extension and NNE-SSW shortening^1^. Eastern China consists mainly of the North China Craton (NCC) in the north and the Yangtze Craton and the Cathaysia Block in the south. The tectonic boundary between the NCC and the Yangtze Craton is the Qinling-Dabie orogen, which was produced by the Triassic collision of the two Precambrian blocks^6^. The lithosphere of Eastern China had experienced a significant modification during the Mesozoic to Cenozoic time^7^,^8^. The subduction of the Paleo-Pacific plate triggered the NCC destruction and induced the widespread back-arc extension and intraplate volcanism in East Asia^9^,^10^.

The Chinese national and provincial seismic networks consist of over 1000 permanent seismic stations, which cover densely most parts of Mainland China (Fig. 2), providing us with an unprecedented opportunity to study the fine-scale structure of the crust and upper mantle. By using the seismic data recorded by these stations as well as temporary seismic arrays deployed on the Tibetan plateau, high-resolution P and S wave velocity tomographic images beneath Mainland China have been obtained^11^,^12^,^13^,^14^,^15^. These results provide important insights into the structure of the subducting Indian, Pacific and Philippine Sea slabs, and the origin of active intraplate volcanism^10^. Seismic velocity tomography is a snapshot of the current structure of the dynamic Earth’s interior, whereas seismic anisotropy can be used to constrain the crustal and mantle deformation and therefore geodynamic processes.

Shear wave splitting (SWS), a phenomenon that an incident shear wave (S-wave) splits into two orthogonal components with different velocities when passing through an anisotropic medium, provides some of the most direct constraints on anisotropy in the Earth and has been widely used to investigate seismic anisotropy in the crust and upper mantle of tectonically active regions^16^. In China, SWS measurements have been made at both regional^17^ and local scales^18^,^19^,^20^. It is generally considered that the observed anisotropy is caused by lithospheric deformation in western China^19^, whereas in eastern China it is attributed to asthenospheric flow controlled by the absolute plate motion (APM) and topography of the lithosphere-asthenosphere boundary^20^.

The SWS measurements are commonly made by using core-refracted phases such as SKS and SKKS. Because the observed splitting time is the integral of anisotropy over the entire steeping ray path (from the core-mantle boundary to the surface), it is hard to discriminate the depth of the anisotropic source, making the interpretation of SWS results not unique and straightforward. In the past decade, there have been significant advances in seismic imaging techniques. The detailed 3-D azimuthal anisotropy structure can be revealed using high-resolution P-wave anisotropic tomography and ambient noise tomography. Thanks to the good vertical resolution of these methods, variations of seismic anisotropy with depth can be revealed, which is essential for clarifying outstanding questions such as the crust-mantle coupling^21^,^22^, lithospheric layering^23^ and subduction dynamics^24^,^25^,^26^,^27^,^28^. Recently, P-wave anisotropic tomography studies have been made to investigate the upper mantle anisotropy beneath Mainland China^29^,^30^. However, due to the limited ray coverage in these studies, only a single anisotropic layer in the upper mantle was assumed in the tomographic inversion^29^ or only the anisotropy in the lithosphere was well resolved^30^. In this study, we present a new model of P-wave anisotropic tomography, which has a high enough resolution in both the lithosphere and asthenosphere, providing new insight into the mantle structure and dynamics of Mainland China. Detailed resolution tests for our tomographic model are made and discussed in Supplementary Information (see Supplementary Figures S1–S6 online).

## Data

**Figure 2:**
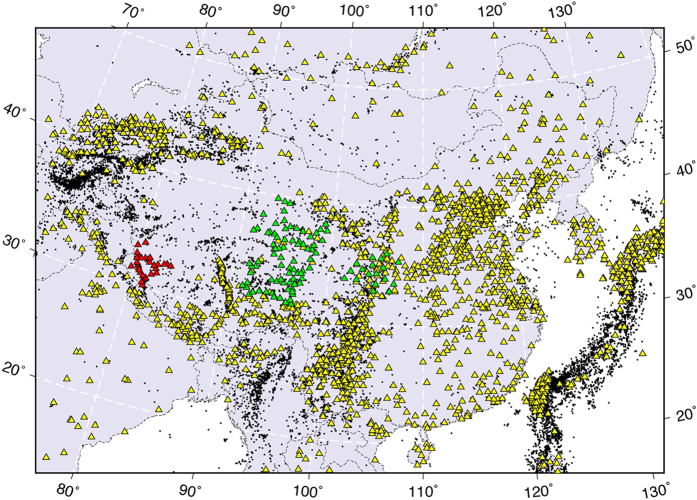
Distribution of earthquakes (black dots) and seismic stations (triangles) used in this study. The green and red triangles denote portable seismic stations deployed by the ASCENT and Western Tibet experiments, respectively. This figure was generated using the Generic Mapping Tools^54^ version 4.5.8 (http://gmt.soest.hawaii.edu).

We used P-wave arrival-time data selected from the following sources: (1) reports of provincial and national seismic networks in China from 1970 to 2006; (2) the EHB bulletin^31^ for the period of 1970–2008; and (3) hand-picked arrival times from the temporary seismic networks deployed in the Tibetan Plateau. We have refined these data sets by removing the overlapped and unreliable records. The combined data set contains more seismic rays than our previous study^13^. In particular, the newly picked arrival-time data from the ASCENT and Western Tibet experiments (Fig. 2) improve the ray coverage in the Tibetan Plateau, enabling us to reveal the fine-scale anisotropic structure there. As a result, we have collected a total of 1,488,531 P-wave arrival times from 14,067 local and regional earthquakes for the present tomographic study (Fig. 2).

## Results

**Figure 3:**
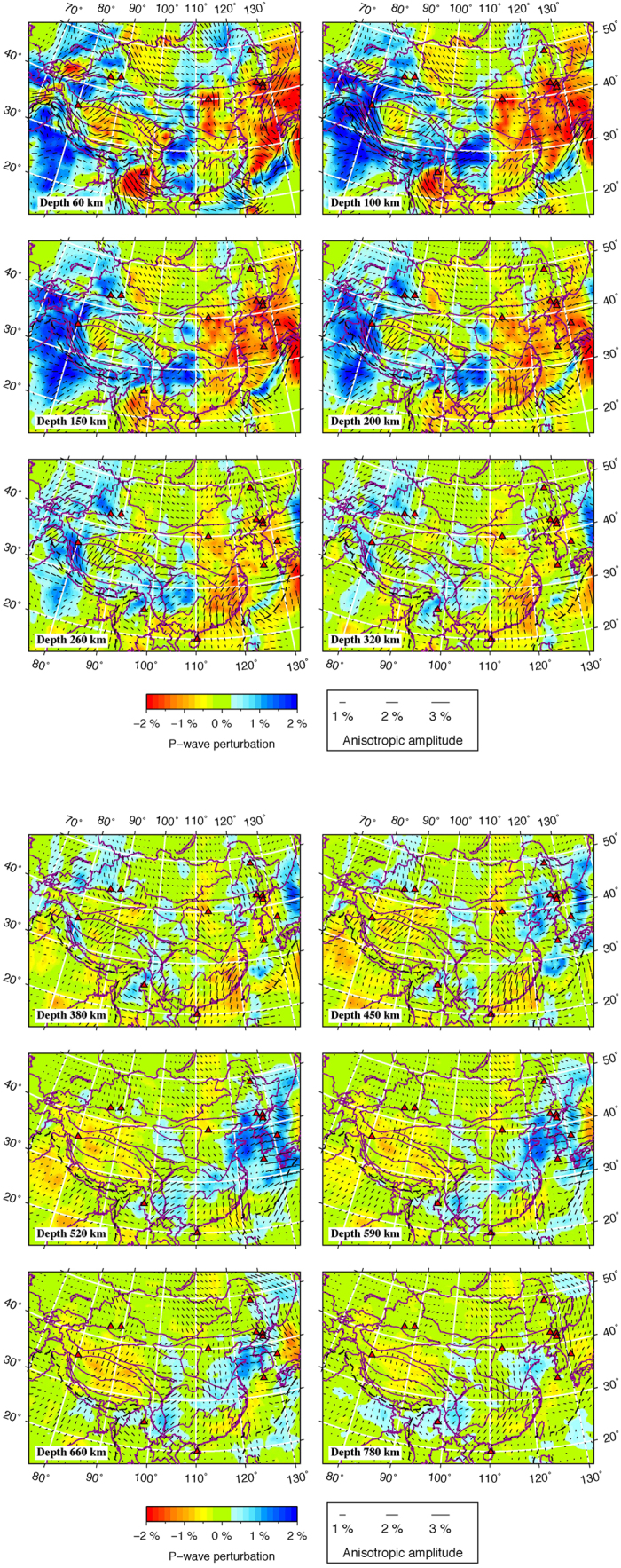
Map views of P-wave anisotropy tomography in China and surrounding areas. The red and blue colors denote slow and fast velocity perturbations, respectively. The orientation and length of each black bar represent fast velocity direction and anisotropic amplitude, respectively. The scales for the isotropic velocity and anisotropic amplitude are shown at the bottom. The red triangles denote active volcanoes. This figure was generated using the Generic Mapping Tools^54^ version 4.5.8 (http://gmt.soest.hawaii.edu).

Figure 3 shows map views of P-wave velocity (Vp) tomography and azimuthal anisotropy in the crust and mantle under the study region. The isotropic Vp images resolved by this study are quite similar to our previous results^13^, although different data sets, grid setting and inversion strategies are adopted, suggesting that our isotropic Vp model is quite robust. One striking feature of our isotropic Vp model is a long-wavelength high-velocity (high-V) anomaly at depths of 60–260 km beneath the Indian shield, the Himalaya and the southern Tibetan Plateau (Fig. 3). The northern limit of this anomaly varies from the west to the east beneath the Tibetan Plateau, suggesting different horizontal extents of the northward subducting Indian plate. This feature has been also identified by recent receiver-function studies^32^,^33^ and tomographic imagings^13^,^14^,^15^. In central Tibet, low-velocity (low-V) anomalies are prominent at depths of 60–200 km, which is consistent with previous tomographic images^12^,^13^,^14^,^15^, possibly reflecting significant variations of rock composition and temperature in the uppermost mantle beneath the southern and central Tibetan Plateau. Our model also reveals high-V mantle roots beneath the Tarim Basin, the Ordos Block and the Yangtze craton, as well as large-scale low-V anomalies beneath eastern China. Thanks to the availability of abundant data recorded by the dense local seismic networks in different parts of Mainland China, the 3-D velocity structure in the study region has been well revealed, and different studies have obtained comparable results^13^,^14^,^15^. However, the 3-D anisotropic structure of this region is still not very clear. Hence, in the following we focus our discussion on depth variations of Vp azimuthal anisotropy in the study region.

A significant variation of FVD is visible in the Indian lithosphere in the depth range of 60–200 km. In general, the FVD is NE-SW under the Indian shield, but it changes to nearly arc parallel under the Himalaya and the Tibetan Plateau. The FVDs in the subducted Indian plate are also roughly parallel with the orientation of the northern limit of the subducting Indian plate beneath the Tibetan Plateau. In the deeper areas, the FVD is NE-SW beneath most parts of the Tibetan Plateau, which is very different from the arc-parallel FVD in the subducted Indian plate as mentioned above. In east of the Burma arc, a dominant NW-SE FVD is observed at depths of 60–150 km, which occurs in a prominent low-V zone and probably reflects corner flow in the mantle wedge due to the active subduction of the Indian plate (or the Burma microplate).

Our results show that the amplitude of azimuthal anisotropy in East China is smaller than that in West China (Fig. 3). The lithospheric thickness in East China is about 100 km^34^, hence the FVDs at depths of 150-200 km shown in Fig. 3 mainly reflect the anisotropy in the asthenosphere. In the NCC, very complex FVDs are revealed at depths of 60 and 100 km, but in deeper areas the FVDs tend to become NW-SE, which is generally parallel to the APM direction. To the south, a predominant NE-SW FVD is revealed throughout the lithosphere of the Yangtze craton, which agrees well with recent results of surface-wave tomography^35^. The upper mantle FVD under Qinling-Dabie is parallel to the strike of the orogenic belt, which may result from the collision between the NCC and the Yangtze craton. In the Cathaysia Block, the anisotropic amplitude exhibits significant lateral variations. It is much stronger in the eastern part than the western part of the region. The FVD in the eastern Cathaysia Block is NE-SW at 100 km depth but gradually changes to NNE-SSW in the depth range of 260–320 km.

## Discussion

In the past two decades, many SKS splitting studies have been made to characterize the azimuthal anisotropy beneath India, Himalaya and the Tibetan Plateau. These studies provide important constraints on the structure and dynamics of the India-Eurasia collision zone. Although some local-scale anisotropy variations have been revealed, the overall fast polarization direction (FPD) in the Indian continent is NNE-SSW^36^,^37^, which is generally parallel with the Indian APM, and the observed anisotropy is attributed to flow in the asthenosphere^36^. To the north along the Himalaya arc, where the collision effects become significant, the measured FPD is rotated to the ENE-WSW direction^37^. In addition, dominant null splitting measurements were also reported^38^, which are mainly located in central Himalaya and the southern Lhasa Terrane. The pattern of anisotropy becomes more complicated in the Tibetan Plateau. As a whole, the SKS splitting results exhibit approximately E-W to NE-SW FPDs in most of central and southern Tibet^18^, whereas they gradually change to NW-SE toward eastern Tibet^19^. According to the variations of SKS splitting delays derived from N-S trending seismic arrays, the leading front of the subducted Indian lithosphere has been inferred^38^.

**Figure 4:**
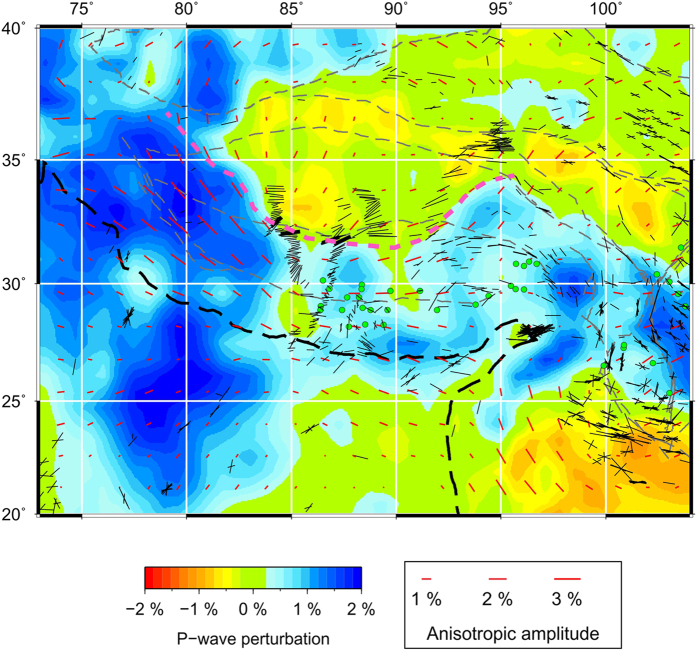
Comparison of shear-wave splitting measurements (black bars, from http://splitting.gm.univ-montp2.fr/DB 55) with P-wave azimuthal anisotropy at 100 km depth revealed by this study (red bars). The northern edge of the subducting Indian plate is marked by the pink dashed line, which is inferred from our P-wave velocity tomography at 200 km depth. The green dotes show the locations of null shear-wave splitting measurements in and around the Tibetan Plateau (from http://splitting.gm.univ-montp2.fr/DB^55^). This figure was generated using the Generic Mapping Tools^54^ version 4.5.8 (http://gmt.soest.hawaii.edu).

**Figure 5:**
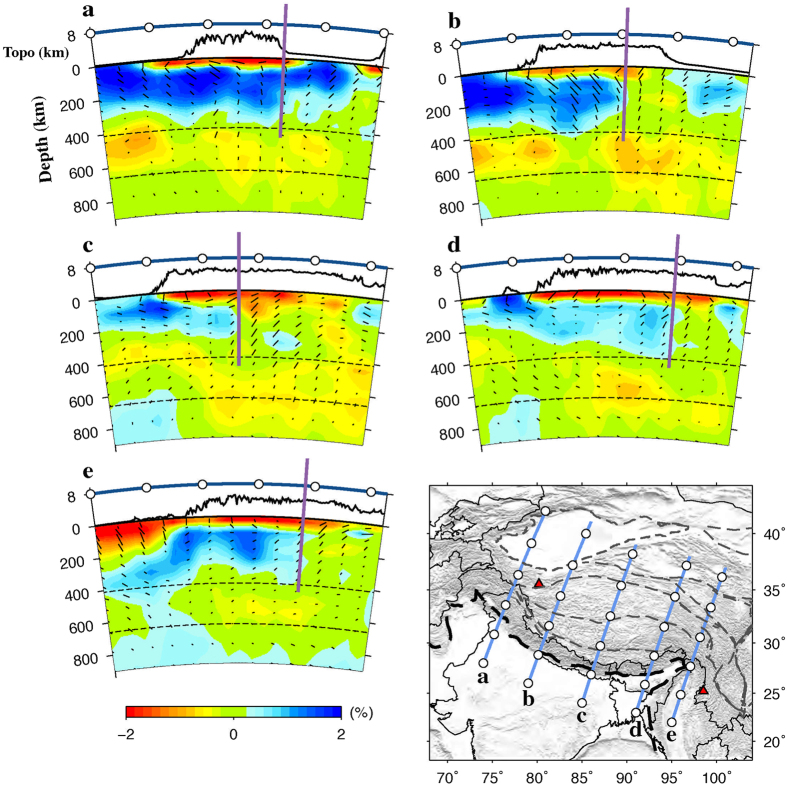
Vertical cross-sections of P-wave anisotropy tomography along 5 profiles shown on the inset map. The thick purple lines show the northern limit of the subducting Indian plate. Note that the orientations of black bars denote the azimuth of FVDs, that is, the vertical bars represent the north-south FVD, whereas the horizontal bars represent the east-west FVD. The other labeling is the same as that in Fig. 3. This figure was generated using the Generic Mapping Tools^54^ version 4.5.8 (http://gmt.soest.hawaii.edu).

**Figure 6:**
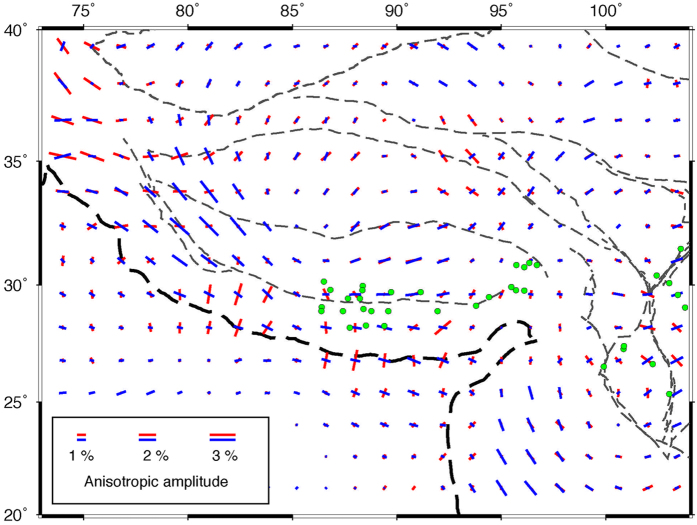
Comparison of P-wave azimuthal anisotropy results in the crust (25 km depth, red bars) and the upper mantle (100 km depth, blue bars). The green dotes show the locations of null shear-wave splitting measurements in and around the Tibetan Plateau. This figure was generated using the Generic Mapping Tools^54^ version 4.5.8 (http://gmt.soest.hawaii.edu).

Our P-wave anisotropy tomography shows a similar pattern of FVDs between 100 and 200 km depths beneath India and the Tibetan Plateau. Figure 4 shows a comparison of the P-wave FVDs at 100 km depth with the SKS splitting measurements. As described above, a significant anisotropy exists in the Indian lithospheric mantle. The FVDs are NE-SW under most of the Indian continent, which are comparable to the SKS observations. Because of the poor vertical resolution of the SKS splitting measurements, it is hard to discriminate where the observed anisotropy exists at depth. Our present results indicate that anisotropy in the lithosphere may be responsible for the SWS observations in the region. Our results also suggest that the NE-SW FVDs in the Indian lithosphere have changed to the direction of arc-parallel when it subducts beneath the Himalaya and the Tibetan Plateau. Following previous studies^32^, we estimated the northern boundary of the Indian lithosphere according to our present Vp tomographic images at 200 km depth (Fig. 4). It is clear that the Indian lithosphere has subducted to the Jinsha River suture in eastern Tibet, farther north than that revealed by previous P-wave tomography^12^. However, our present result is consistent with recent P- and S-wave tomography^13^,^14^,^15^. This feature is also visible in vertical cross-sections passing through the Tibetan Plateau (Fig. 5). The northern edge of the subducting Indian plate is generally parallel with the India-Asia plate boundary at the surface, and the FVDs in the area between them are also roughly parallel with the plate boundary (Fig. 4). These FVDs are, to the first order, correlated with the SKS splitting results (Fig. 4), though there are some differences especially in eastern Tibet where dominant NW-SE FPDs are detected. These FPDs, however, generally agree with the resolved P-wave azimuthal anisotropy in the crust (Fig. 6), suggesting that the crustal anisotropy has contributed significantly to the SKS splitting measurements in the region.

Our present results provide strong evidence for the depth-dependent anisotropy beneath the Tibetan Plateau and surrounding regions. Figure 6 shows a comparison of the observed azimuthal anisotropy at 25 and 100 km depths. A variation of anisotropy with depth is clearly visible beneath the western and southeastern Tibetan margins and nearly the whole Himalaya (Fig. 6). Previous SKS splitting studies found that null splitting measurements are dominant in the Himalaya and some parts of the Tibetan Plateau^33^,^38^. However, the origin of these null measurements is still unclear. Our present results show clearly that these null measurements are mainly distributed in regions where two anisotropic layers exist (Fig. 6). It should be noted that the anisotropy is also present in the sublithospheric mantle and even in the mantle transition zone (Fig. 3), suggesting that more anisotropic layers could be present in the region. Therefore, we attribute these null measurements to the joint effects of two or more layers with different anisotropic properties.

As mentioned above, the FVDs in the Indian lithosphere have changed from NE-SW under the Indian continent to arc parallel under the Himalaya and the Tibetan Plateau. This result suggests that the frozen orientations in the Indian lithosphere during the post-tectonic thermal relaxation have been “erased” and the minerals have been re-oriented along the direction of extension due to the most recent tectonic activities of the India-Eurasia collision. Results of SWS measurements suggest that striking variations in the FPD and delay time exist in the Tibetan Plateau. There is no a simple model that can explain these complex splitting results. However, our present tomographic results suggest that the most likely explanation is a combination of anisotropy in the crust due to crustal ductile flow, that in the subducted Indian lithosphere, and a contribution of anisotropy in the sublithospheric mantle and the mantle transition zone.

Recent results of SWS measurements demonstrate that the main FPDs are NW-SE in the eastern part of North China^17^,^20^, which are roughly parallel to the absolute motion direction of the Pacific plate and are likely caused by asthenospheric flow due to the plate motion^17^. To the south, the FPDs are aligned in the direction of ENE-WSW in the Cathaysia Block, which are significantly different from the APM direction, suggesting that the mantle flow is deflected by the thick lithospheric mantle roots^20^.

Our present tomography shows different mantle structures beneath the western and eastern parts of the NCC. In the western NCC, high-V lithospheric roots are clearly revealed to a depth of at least 260 km, whereas in the eastern NCC, dominant low-V anomalies are found at these depths. These results support the idea that the thick cratonic lithosphere in the eastern NCC was destroyed during the Mesozoic-Cenozoic NCC reactivation, as revealed by many geochemical and petrological studies^7^,^8^. Our results show a complex pattern of azimuthal anisotropy at 60 km depth under the eastern NCC: the FVDs are mainly trending NE-SW to E-W in the southern part but N-S in the northern part. Such a pattern most likely reflects the fossil anisotropy in the lithosphere, caused by the collision of the NCC with the South China Block^39^ and the NCC reactivation. The lithospheric thickness in the eastern NCC is ~70 km^40^, hence the observed NW-SE FVDs below 100 km depth reflect the anisotropy in the asthenosphere. Following many previous studies^17^,^20^,^41^, we attribute the APM-parallel FVDs to the lattice-preferred orientation (LPO) of olivine associated with the asthenospheric flow, caused by the subduction of the Pacific plate^10^.

**Figure 7:**
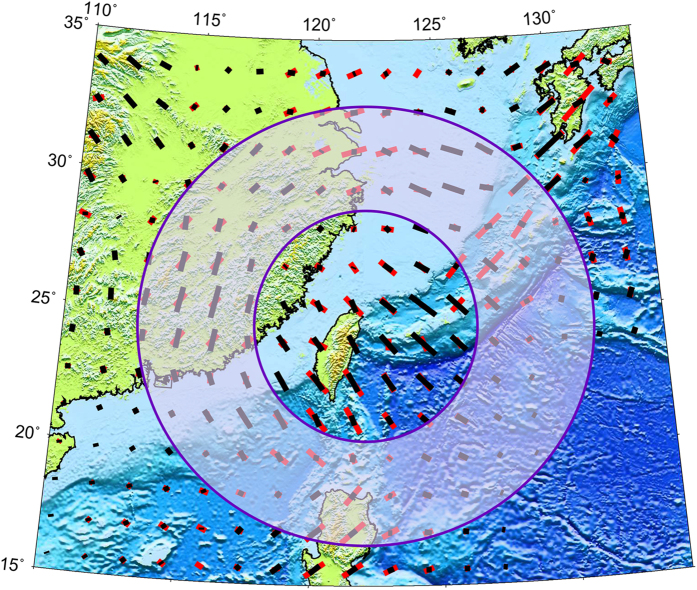
A schematic diagram showing the toroidal flow around the subducting Philippine Sea slab. The red and black bars show the P-wave azimuthal anisotropy at 100 km and 200 km depths, respectively. This figure was generated using the Generic Mapping Tools^54^ version 4.5.8 (http://gmt.soest.hawaii.edu).

The Philippine Sea (PHS) plate is subducting beneath the Eurasian plate northwestward at a rate of ~5.5 cm/yr from the Ryukyu trench^42^. The subducting PHS slab is clearly imaged by recent P- and S-wave velocity tomography^28^ and our present P-wave anisotropy tomography. A significant high-V anomaly corresponding to the subducted PHS slab is visible to a depth of at least 260 km (Fig. 3). More interestingly, a circular pattern of FVDs is revealed around the PHS slab (Fig. 7). Numerical modeling studies suggest that a toroidal flow could be driven around the slab edge due to rollback of the subducting slab^43^,^44^,^45^ or complexity in slab shape^46^. The curved geometry and possible rollback of the subducting PHS slab due to trench retreat^47^ may have caused the toroidal flow around the slab. This pattern of azimuthal anisotropy revealed by our P-wave anisotropy tomography is consistent with the toroidal flow around the PHS slab edge beneath Southeast China and surrounding areas, assuming A-type LPO of olivine in the upper mantle.

## Methods

We adopted the P-wave anisotropic tomographic method^25^,^48^ to invert the selected P-wave arrival times for the 3-D Vp anisotropic structure beneath Mainland China and surrounding regions. Assuming a simple case of weak anisotropy with a horizontal hexagonal symmetry axis, the Pn wave slowness *S*(*ϕ*) (the reciprocal of velocity) can be approximately expressed as follows^49^:


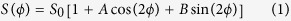


where *S*_0_ is the azimuthal average slowness, *ϕ* is the ray azimuth of a horizontally-propagating Pn wave, *A* and *B* are two azimuthal anisotropy parameters. Equation (1) has been modified^24^ to adapt to a general P-wave propagating at any incident angle *i*:


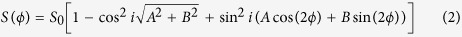


The theoretical travel time from the *j*th event to the *i*th station can be expressed as:


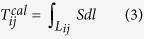


where *L*_*ij*_ is the ray path. A travel-time residue *r* can be written as^24^,^25^,^26^:


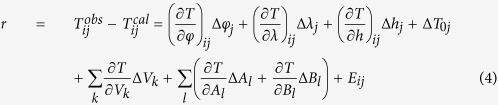


where 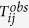 is the observed travel time; *φ*_*j*_, *λ*_*j*_, *h*_*j*_, *T*_0*j*_ are the latitude, longitude, focal depth and origin time of the *j*th event. We set up two 3-D grid nets to express the Vp isotropic and anisotropic structures, respectively. *V*_*k*_ is the isotropic velocity at the *k*th grid node of the first fine grid, whereas *A*_*l*_ and *B*_*l*_ are anisotropic parameters at the *l*th grid node of the second coarse grid. *E*_*ij*_ represents the higher-order terms and errors of the observations.

Following a previous tomographic study^22^, we add the following constraints as damping and smoothing regularizations to the tomographic inversion:


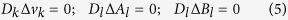



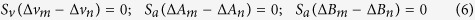


where *D*_*k*_ and *D*_*l*_ are damping parameters for the isotropic Vp and anisotropy, respectively; *S*_*v*_ and *S*_*a*_ are smoothing parameters for the isotropic Vp and anisotropy, respectively; *m* and *n* represent the adjacent grid nodes of the same orientation. We used a conjugate-gradient type solver, the LSQR algorithm^50^, to solve the equations (4, 5, 6). When the anisotropic parameters *A* and *B* are determined, the anisotropic amplitude *α* and the fast velocity direction (FVD) *ψ* of P-wave azimuthal anisotropy can be calculated as follows:


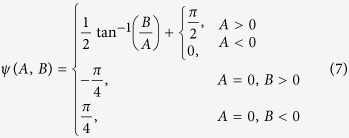



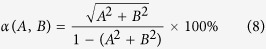


The one-dimensional (1-D) *ak135* model^51^ is adopted as the initial P-wave velocity model for the tomographic inversion. A dense 3-D grid with a horizontal grid interval of 0.7° and a vertical grid interval of 35–150 km is set up in the study region to express the 3-D isotropic Vp structure, and a coarse 3-D grid with a horizontal grid interval of 1.4° is set up to express the 3-D Vp azimuthal anisotropy. Meshes of grid nodes are set up at depths of 25, 60, 100, 150, 200, 260, 320, 380, 450, 520, 590, 660, 780, 900, 1050 and 1200 km. After many synthetic tests and real data inversions, our final tomographic images are obtained by choosing *S*_*v*_ = 200, *S*_*a*_ = 100, *D*_*k*_ = 100 and *D*_*l*_ = 50.

In the study region, the crustal thickness exhibits significant lateral variations. A simple 1-D crustal model adopted may cause significant smearing in the final images. To examine the robustness of our tomographic images in the mantle, we also conducted tomographic inversions by using a starting velocity model which is composed of a 3-D crustal model^52^ and the *ak135* model for the upper mantle. The obtained tomographic results are shown in Supplementary Figure S7. By comparing Fig. 3 and Supplementary Figure S7, it is clear that although there are some differences, the dominant Vp isotropic and anisotropic images in the mantle are quite similar, suggesting that our tomographic results are reliable and robust.

## Additional Information

**How to cite this article**: Wei, W. *et al*. Depth variations of P-wave azimuthal anisotropy beneath Mainland China. *Sci. Rep*. **6**, 29614; doi: 10.1038/srep29614 (2016).

## Supplementary Material

Supplementary Information

## Figures and Tables

**Figure 1:**
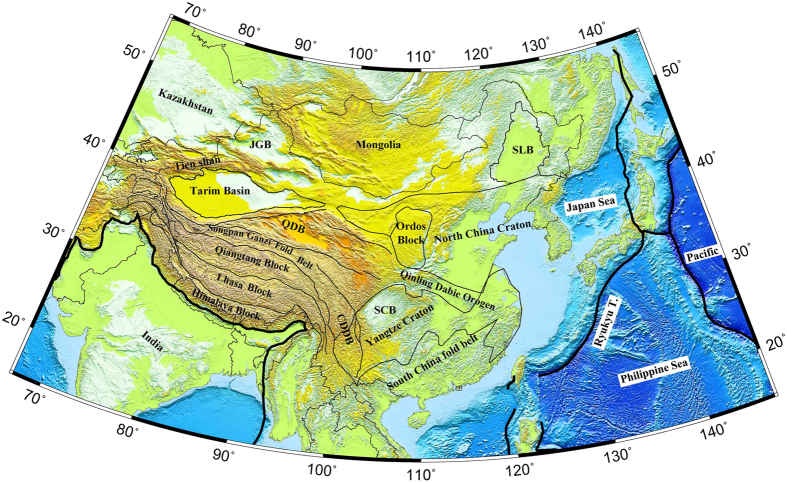
A map showing the surface topography and main tectonic units in China and surrounding areas. The black lines show the major plate boundaries^53^, and the grey lines represent the sutures and main tectonic boundaries. The abbreviations are as follows: CDDB, the Chuandian Diamond Block; SCB, the Sichuan Basin; QDB, the Qaidam Basin; JGB, the Jungger Basin; SLB, the Songliao Basin. This figure was generated using the Generic Mapping Tools^54^ version 4.5.8 (http://gmt.soest.hawaii.edu).
