# Supplementary material for: Depth variations of P-wave azimuthal anisotropy beneath Mainland China
Source: Sci Rep. 2016 Jul 19;6:29614. doi: 10.1038/srep29614 (PMC4949423; doi:10.1038/srep29614)
Supplement: Supplementary Information [file srep29614-s1.pdf]

Depth variations of P-wave azimuthal anisotropy beneath Mainland China – Supplementary information

Wei Wei[1,2,*], Dapeng Zhao[2], Jiandong Xu[1], Bengang Zhou[1] & Yaolin Shi[3]

1 Key Laboratory of Active Tectonics and Volcano, Institute of Geology, China Earthquake Administration, Beijing 100029, China

2 Department of Geophysics, Tohoku University, Sendai 980-8578, Japan

3 Key Laboratory of Computational Geodynamics, Chinese Academy of Sciences, Beijing 100049, China

weiwei25011@gmail.com

ABSTRACT

Supplementary information

Resolution tests

To examine the resolution scale of the present data set and the robustness of the obtained tomography model, we conducted many checkerboard resolution tests (CRT) with different grid intervals. In the CRT, positive and negative velocity and anisotropic parameter perturbations of 2% were assigned alternatively to the grid nodes in both the lateral and vertical directions. Synthetic arrival times were calculated for the checkerboard model with the same source-receiver geometry as for the observed data, and random noise with a standard deviation of 0.45 s was added to the synthetic data. Then we inverted the synthetic data with the same algorithm as that

for the real data to investigate the degree of recovery for both isotropic and anisotropic P-wave velocity (Vp) structures.

One of the CRT results is shown in Figures S1 and S2 which are the recovered images of the isotropic Vp and azimuthal anisotropy with a lateral grid interval of 1.4° and 2.0°, respectively. The test results show that the isotropic Vp anomalies are well resolved for most of the study region down to a depth of 780 km (Figure S1), whereas those under Northeast China are slightly smeared, probably due to the limited ray coverage there. To obtain a reliable Vp anisotropy structure, we need a good azimuthal ray coverage in the modeling space. The CRT results suggest that the input anisotropic features are well retrieved in most parts of the study region, although significant smearing occurs in Northeast China and Inner Mongolia (Figure S2). To further investigate a possible trade-off between the isotropic and anisotropic Vp structures, we made synthetic arrival times for an isotropic Vp model and then inverted the synthetic data for both isotropic and anisotropic Vp structures. The inversion results indicate that the input Vp model is recovered very well (Figure S3), but some artificial anisotropic structures also show up (Figure S4). However, the FVDs of the artificial anisotropic structures are randomly distributed and their amplitudes are generally much smaller than those of azimuthal anisotropy (2-3%) obtained by the real data inversion. Similarly, we inverted synthetic arrival times generated for an anisotropic structure to map both isotropic and anisotropic Vp structures. The results show that the isotropic and anisotropic features are well distinguished (Figures S5 and S6). These synthetic tests suggest the robustness of our

data sets and the tomographic method which can well distinguish the isotropic and anisotropic Vp structures in the study region.

Additional information

The authors declare no competing financial interests.

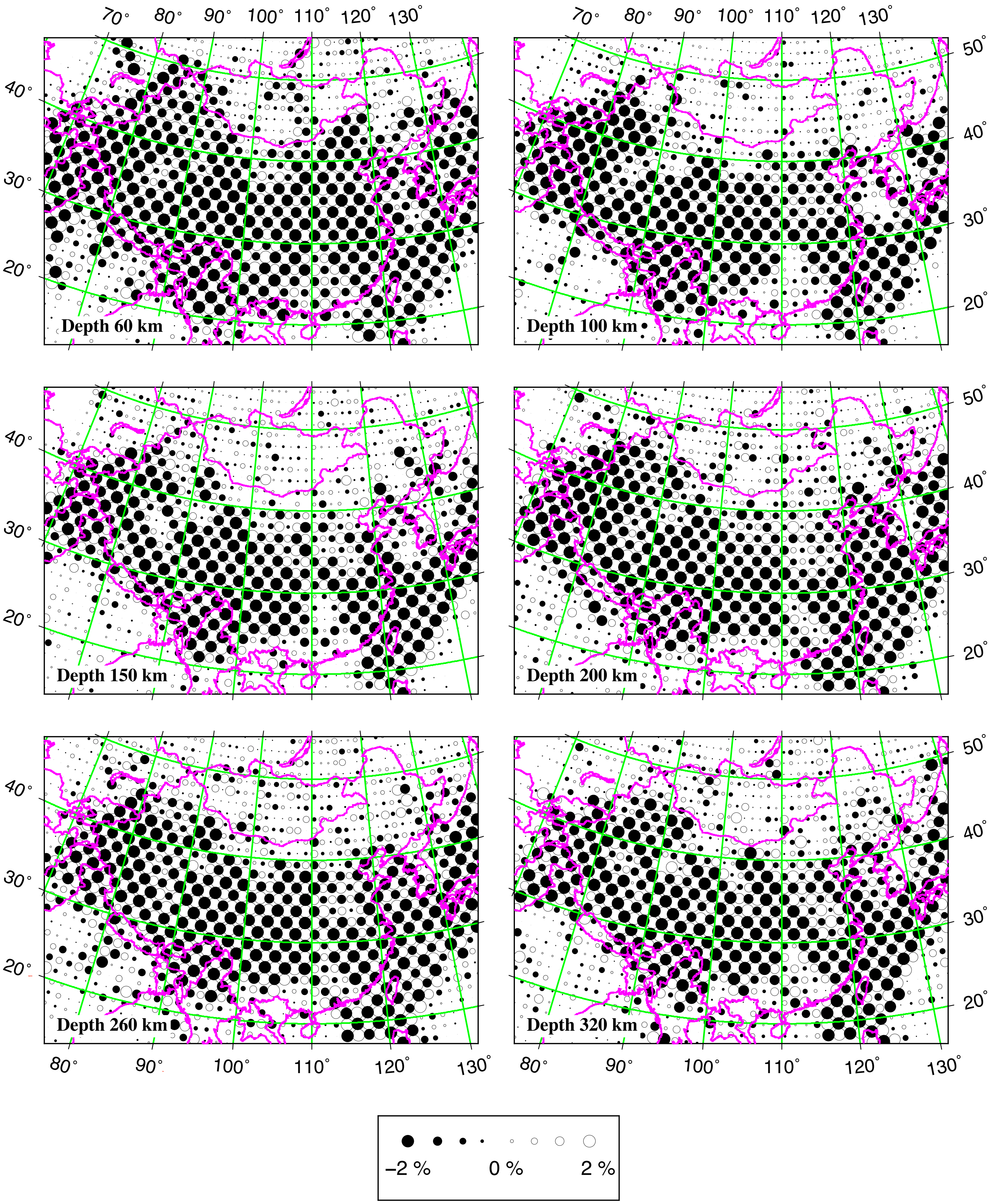

Figure S1. Results of a checkerboard resolution test for P-wave tomography at 12 representative depths. The solid and open circles denote low and high velocities, respectively. The velocity perturbation (in %) scale is shown at the bottom. This figure was generated using the Generic Mapping Tools version 4.5.8 (http://gmt.soest.hawaii.edu)

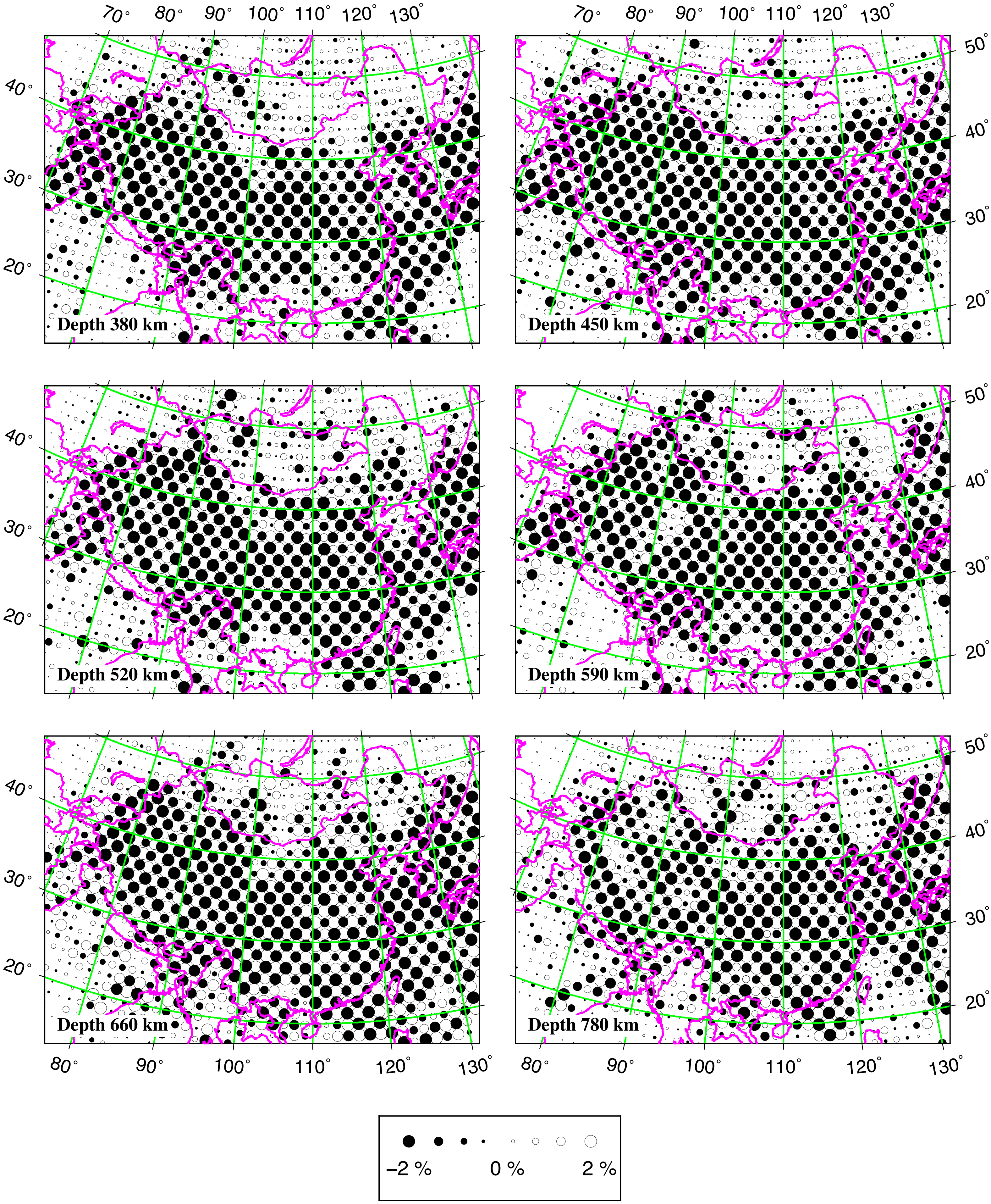

Figure S1 (continued)

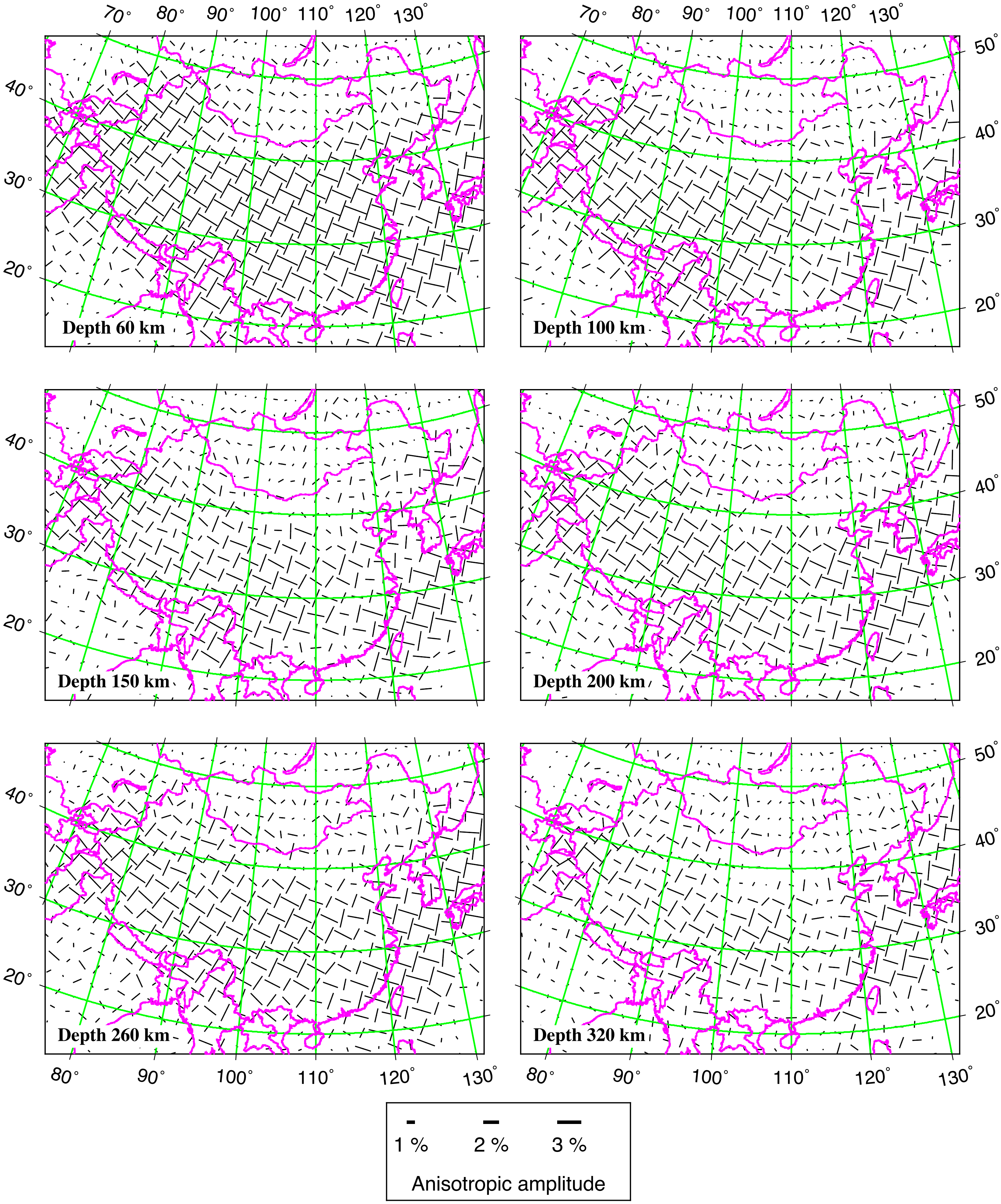

Figure S2. Results of a checkerboard resolution test for P-wave azimuthal anisotropy at 12 depths. The input FVDs at two adjacent grid nodes are perpendicular to each other with the same amplitude of 2.83%. The scale of the anisotropic amplitude is shown at the bottom. This figure was generated using the Generic Mapping Tools version 4.5.8 (http:/gmt.soest.hawaii.edu)

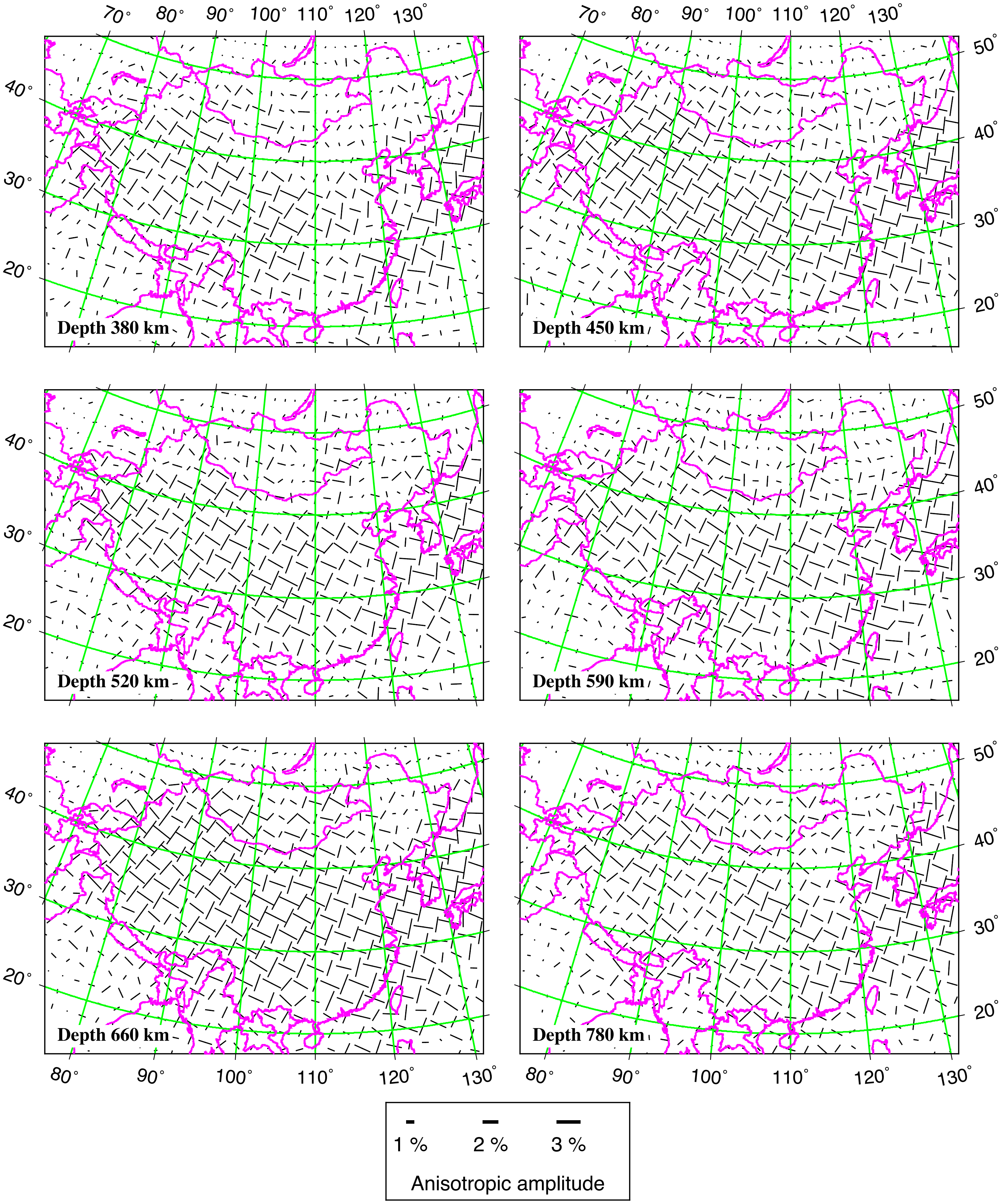

Figure S2 (continued)

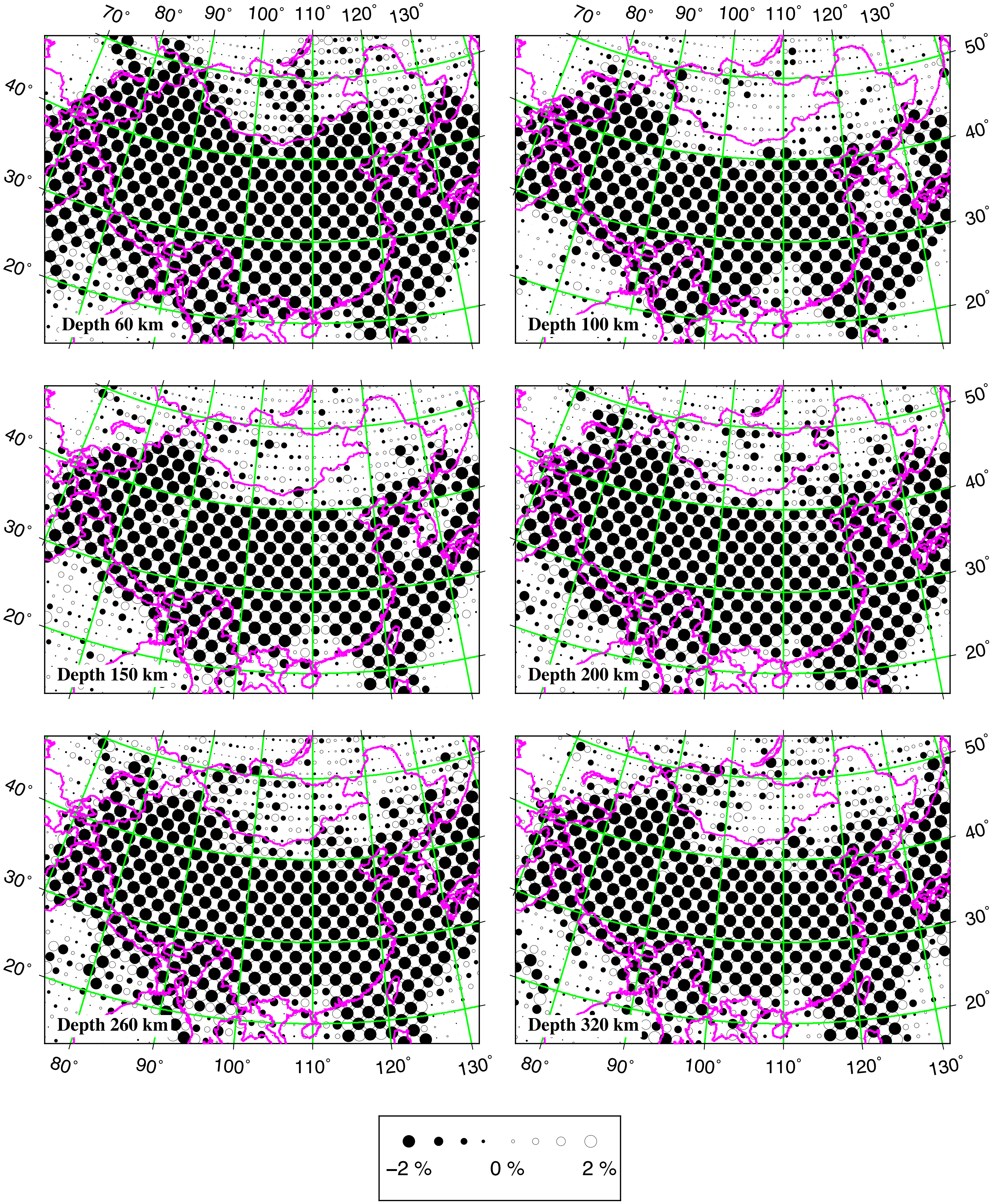

Figure S3. Results of a synthetic resolution test for P-wave velocity structure at 12 depths. The input synthetic model contains only the isotropic Vp structure. This figure was generated using the Generic Mapping Tools version 4.5.8 (http://gmt.soest.hawaii.edu)

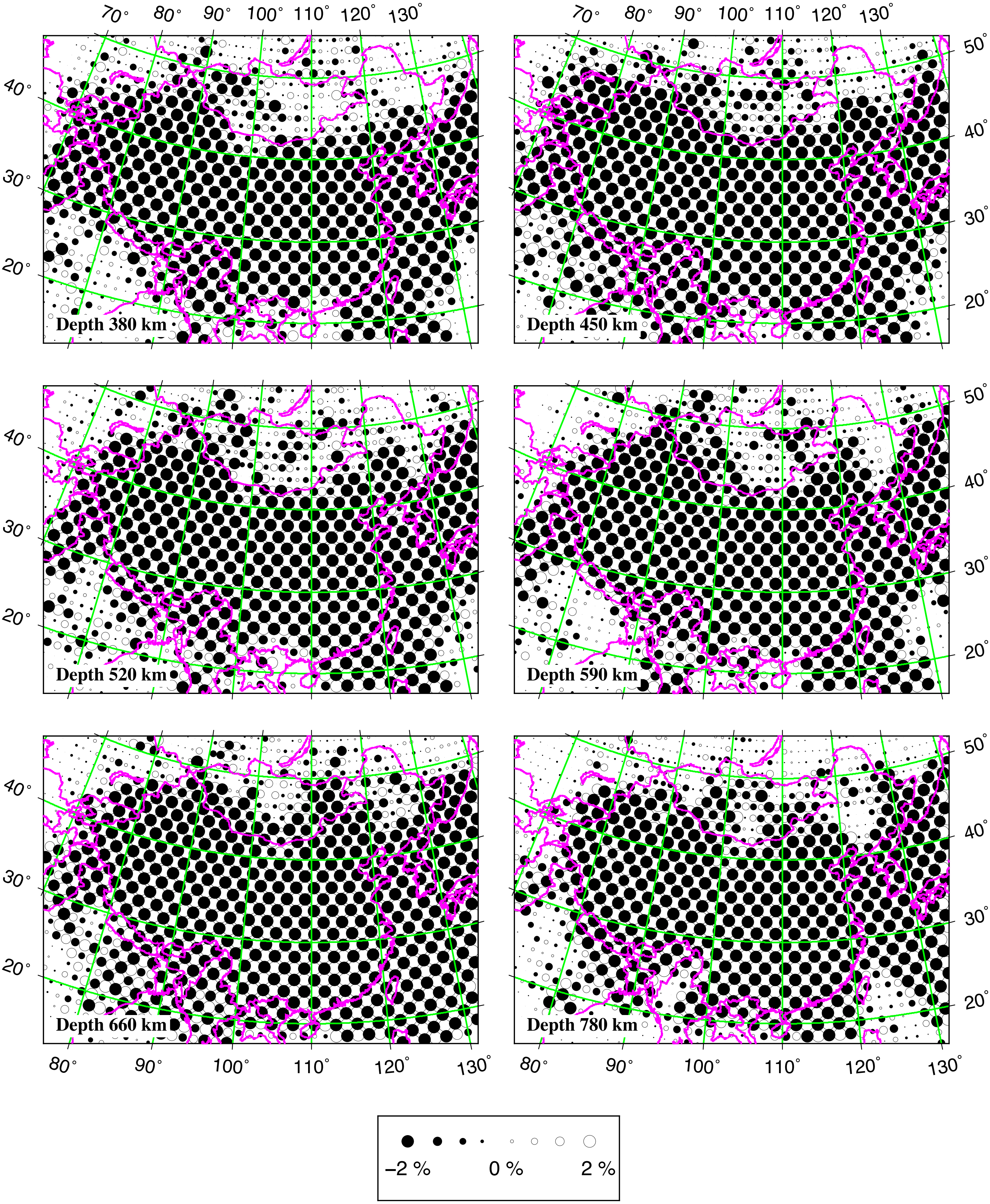

Figure S3 (continued)

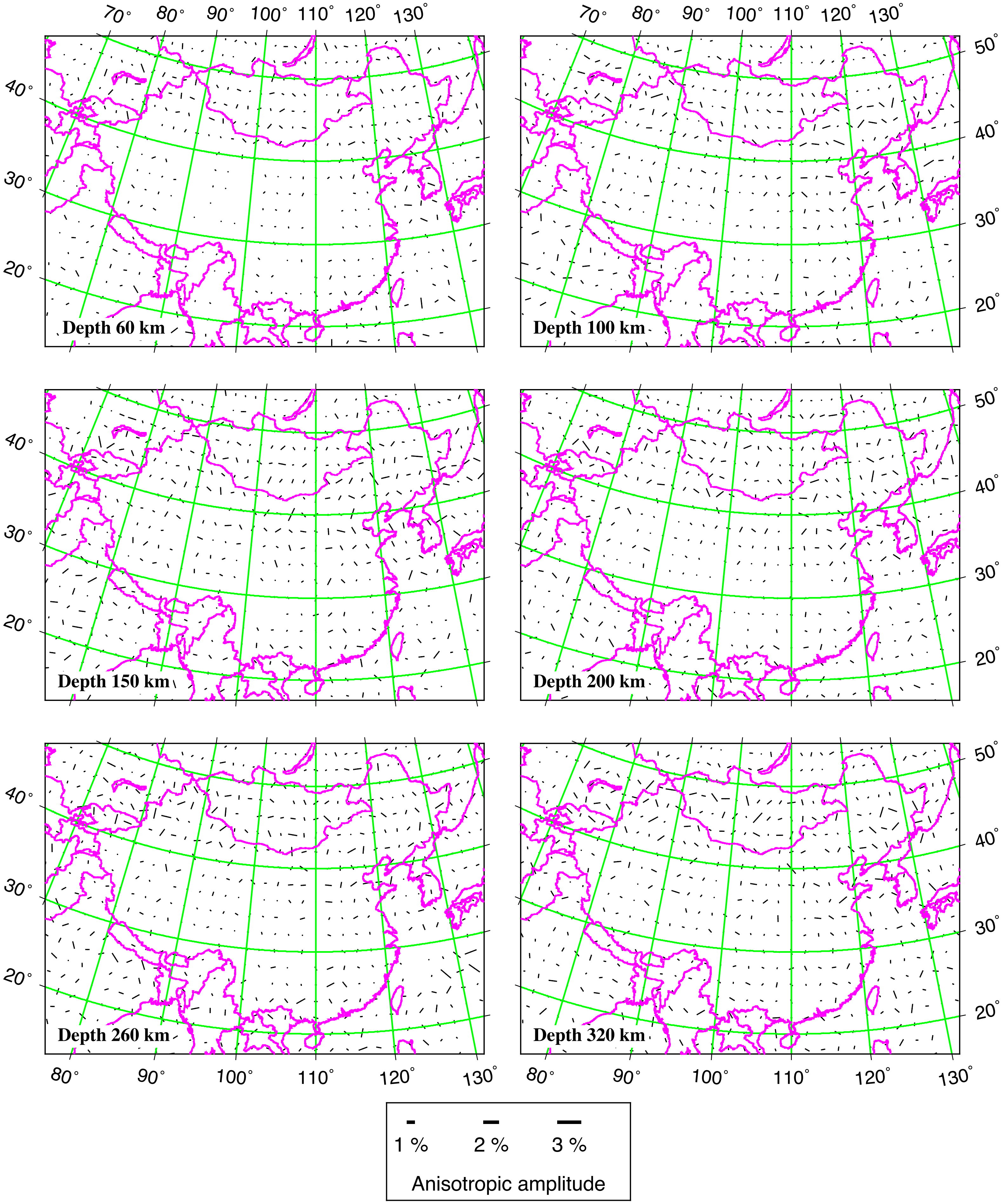

Figure S4. Results of a synthetic resolution test for P-wave azimuthal anisotropy at 12 depths. The input synthetic model contains only the isotropic Vp structure. This figure was generated using the Generic Mapping Tools version 4.5.8 (http://gmt.soest.hawaii.edu)

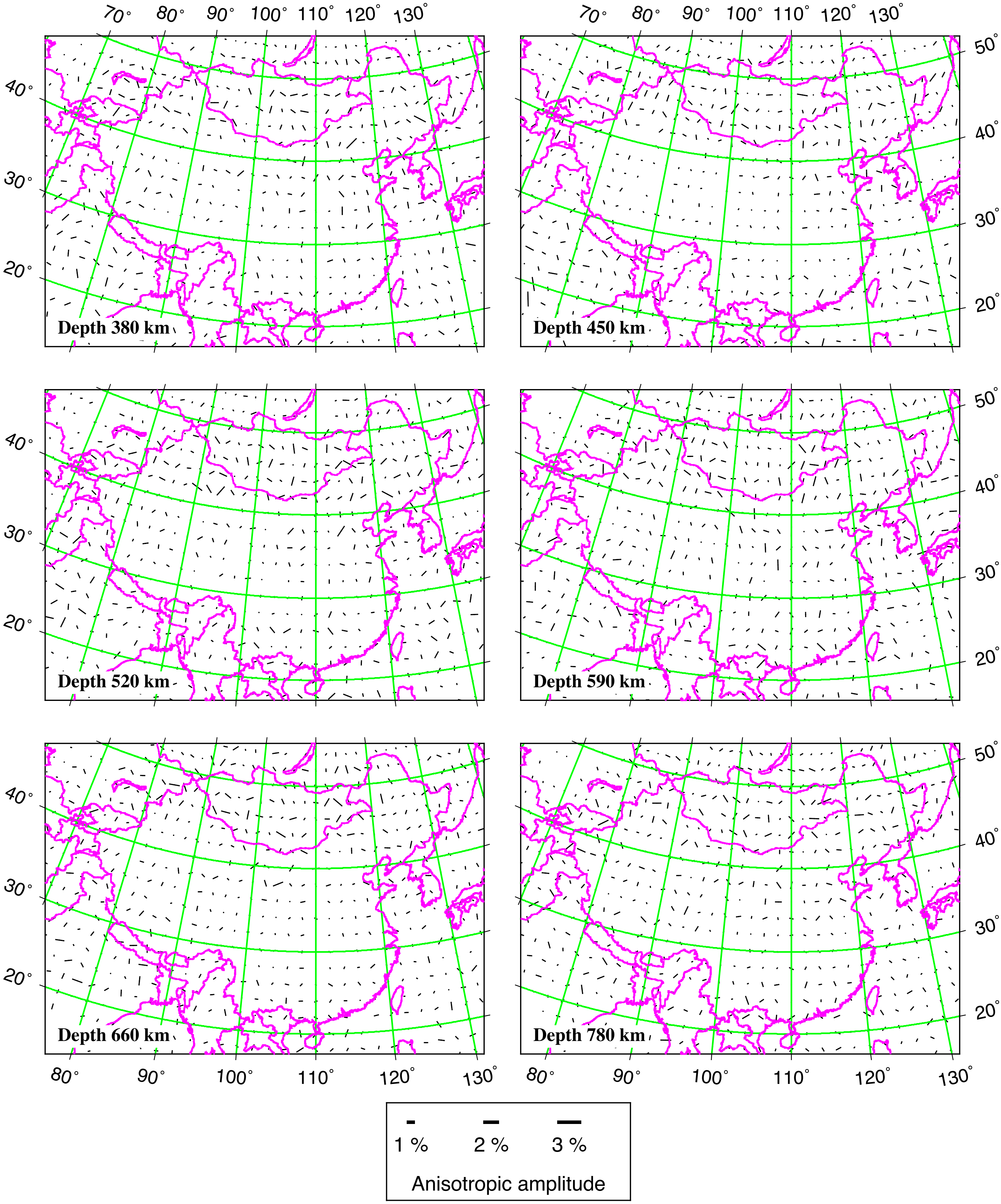

Figure S4 (continued)

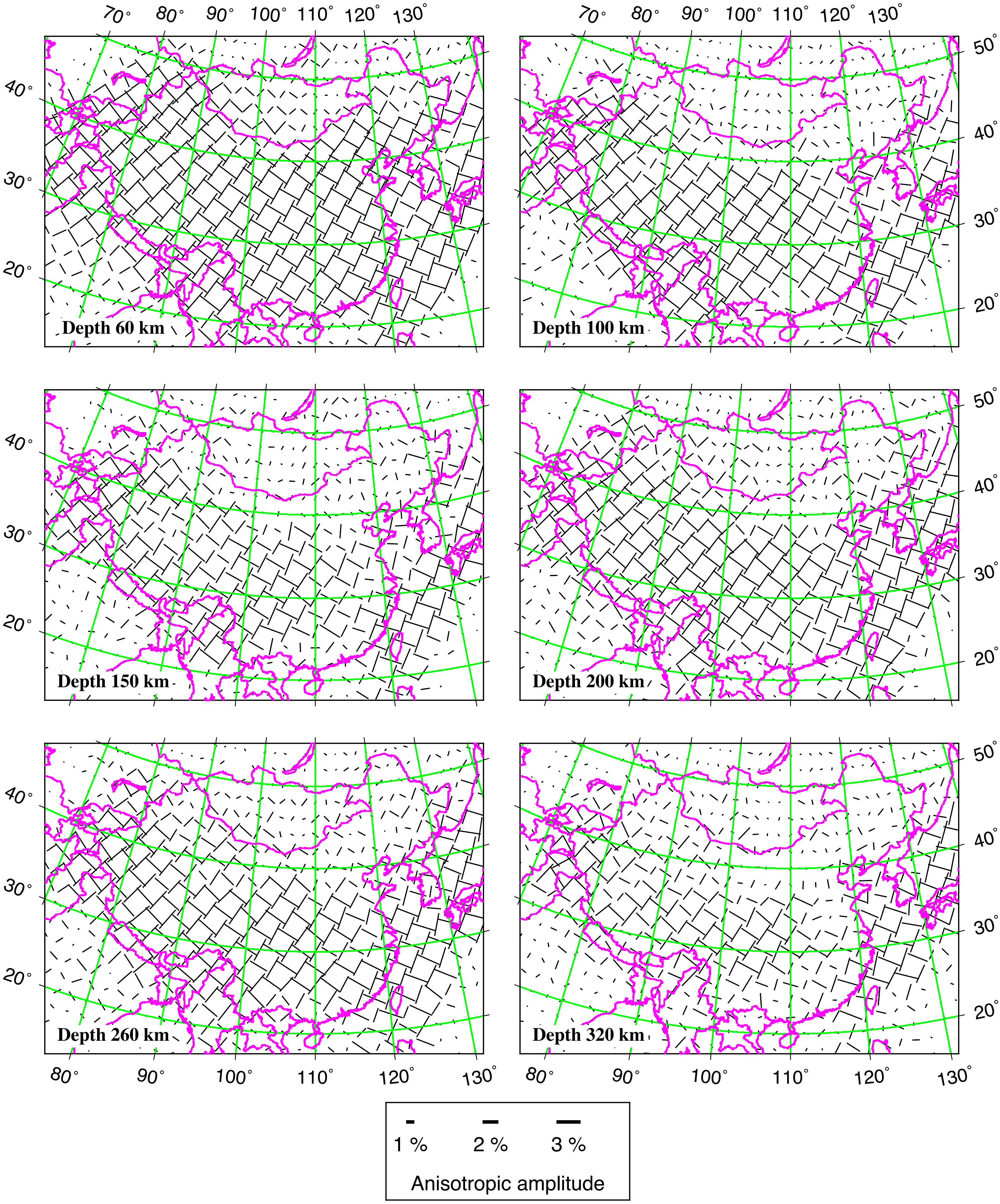

Figure S5. Results of a synthetic resolution test for P-wave azimuthal anisotropy at 12 depths. The input synthetic model contains only the anisotropic Vp structure. This figure was generated using the Generic Mapping Tools version 4.5.8 (http://gmt.soest.hawaii.edu)

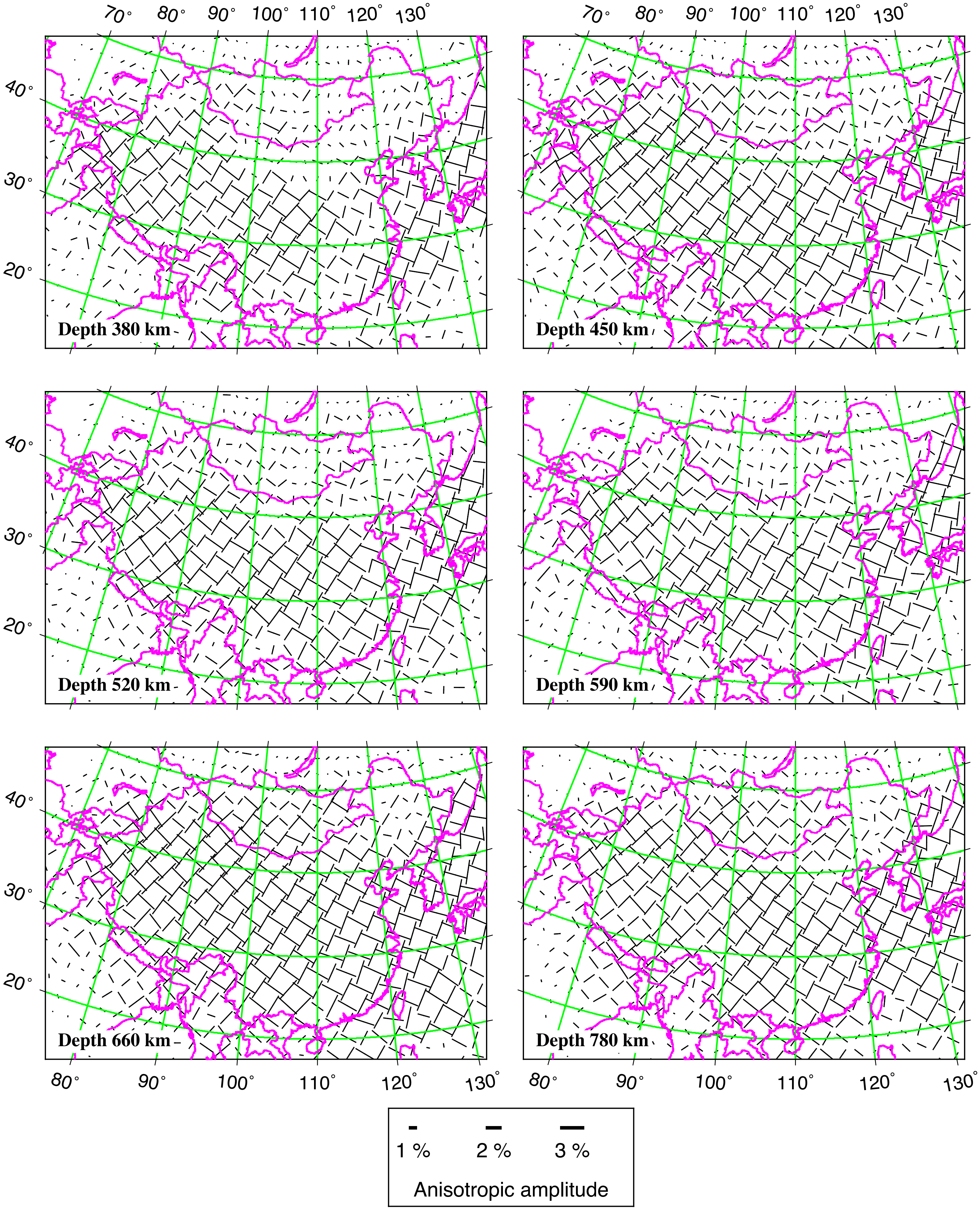

Figure S5 (continued)

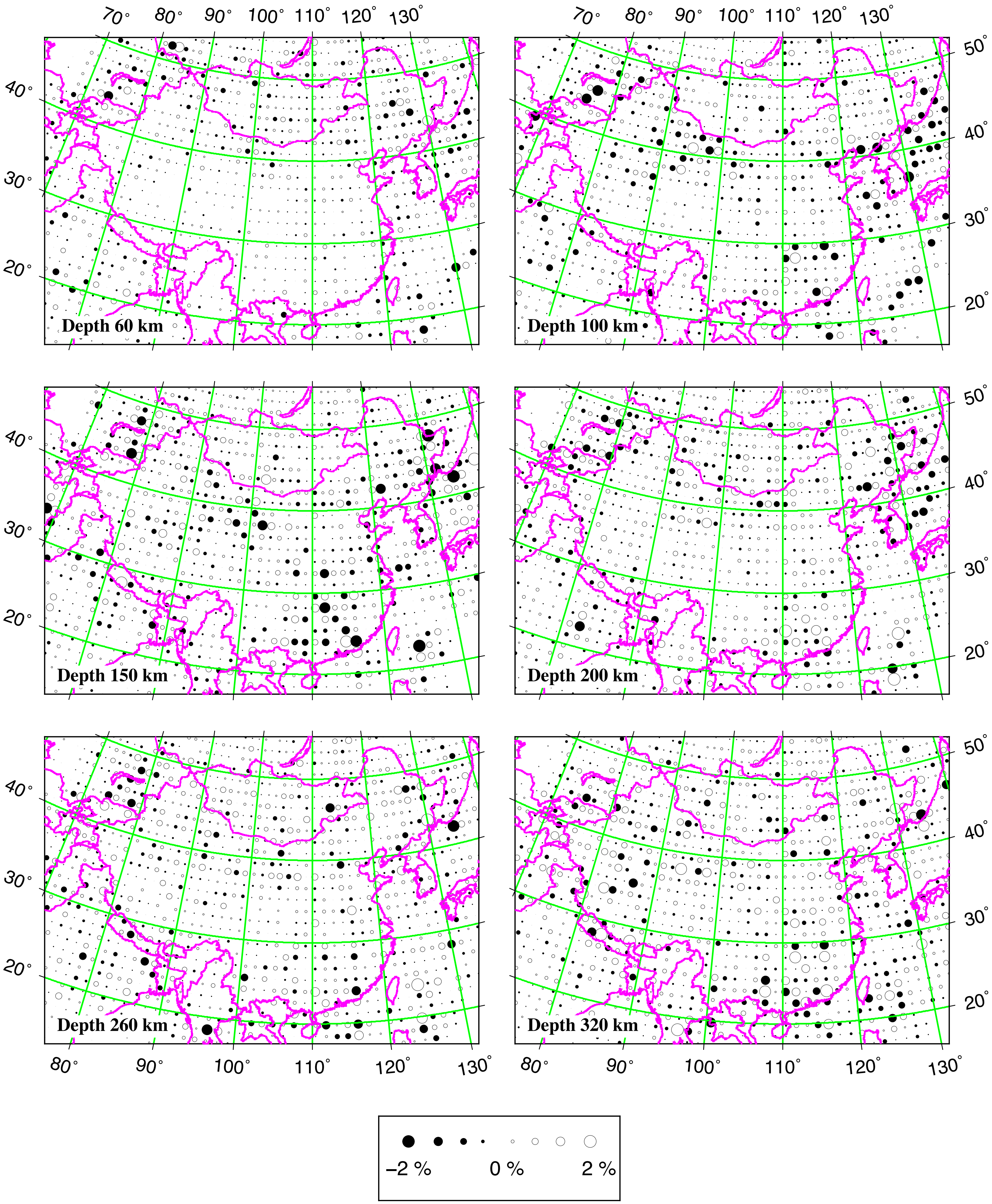

Figure S6. Results of a synthetic resolution test for P-wave velocity structure at 12 depths. The input synthetic model contains only the anisotropic Vp structure. This figure was generated using the Generic Mapping Tools version 4.5.8 (http://gmt.soest.hawaii.edu)

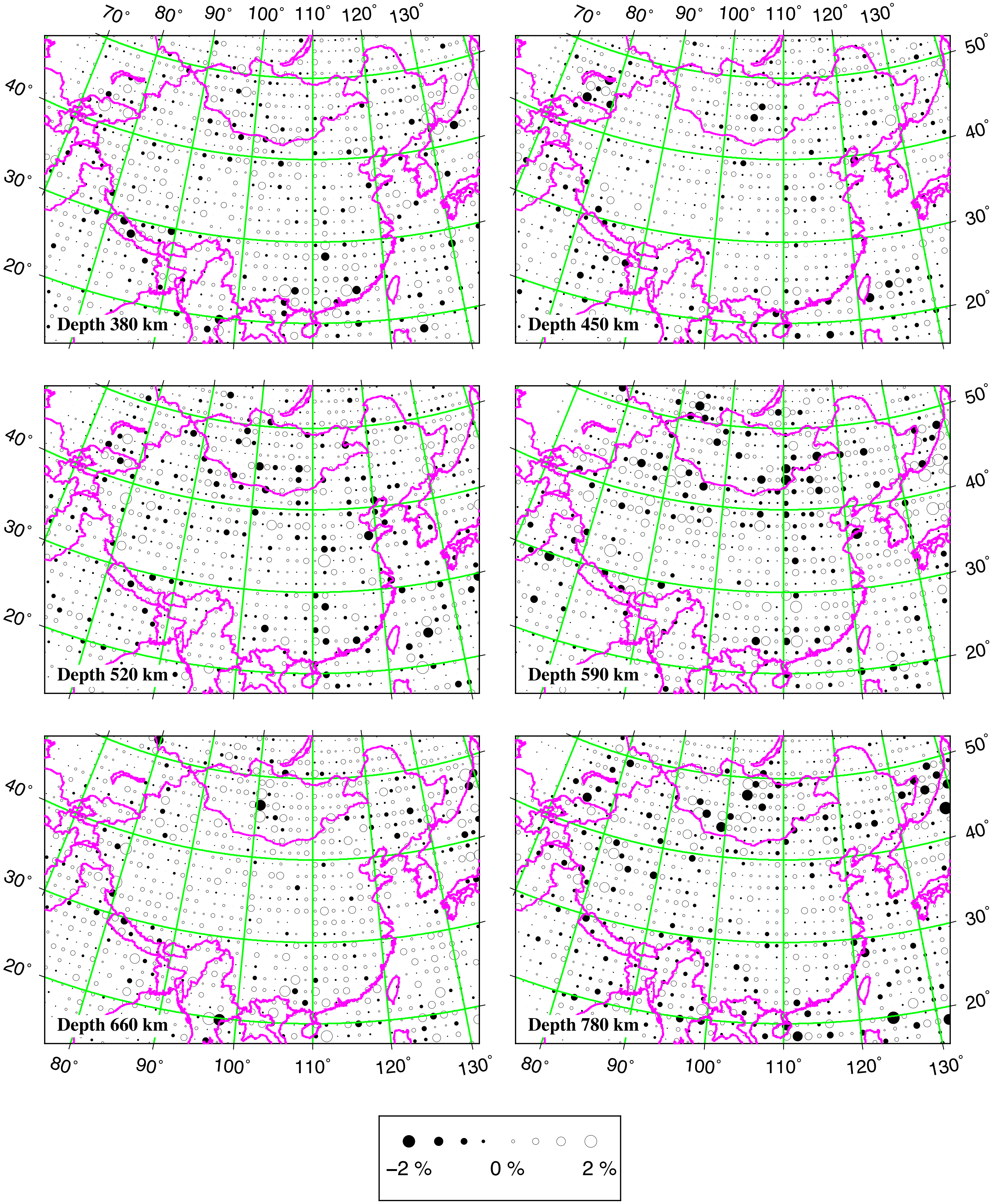

Figure S6 (continued)

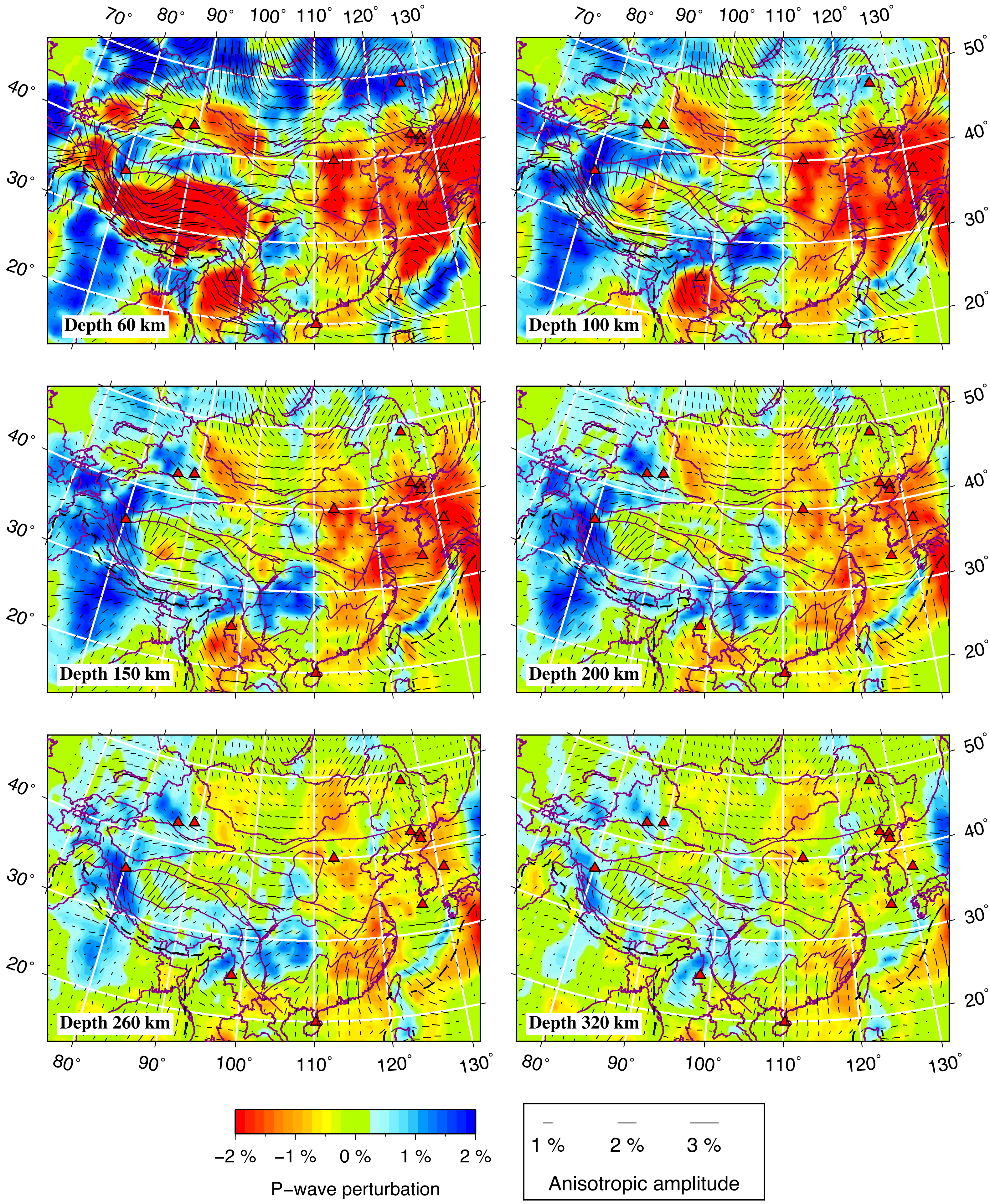

Figure S7. The same as Figure 3 but for tomographic results with a 3-D starting model. This figure was generated using the Generic Mapping Tools version 4.5.8 (http://gmt.soest.hawaii.edu)

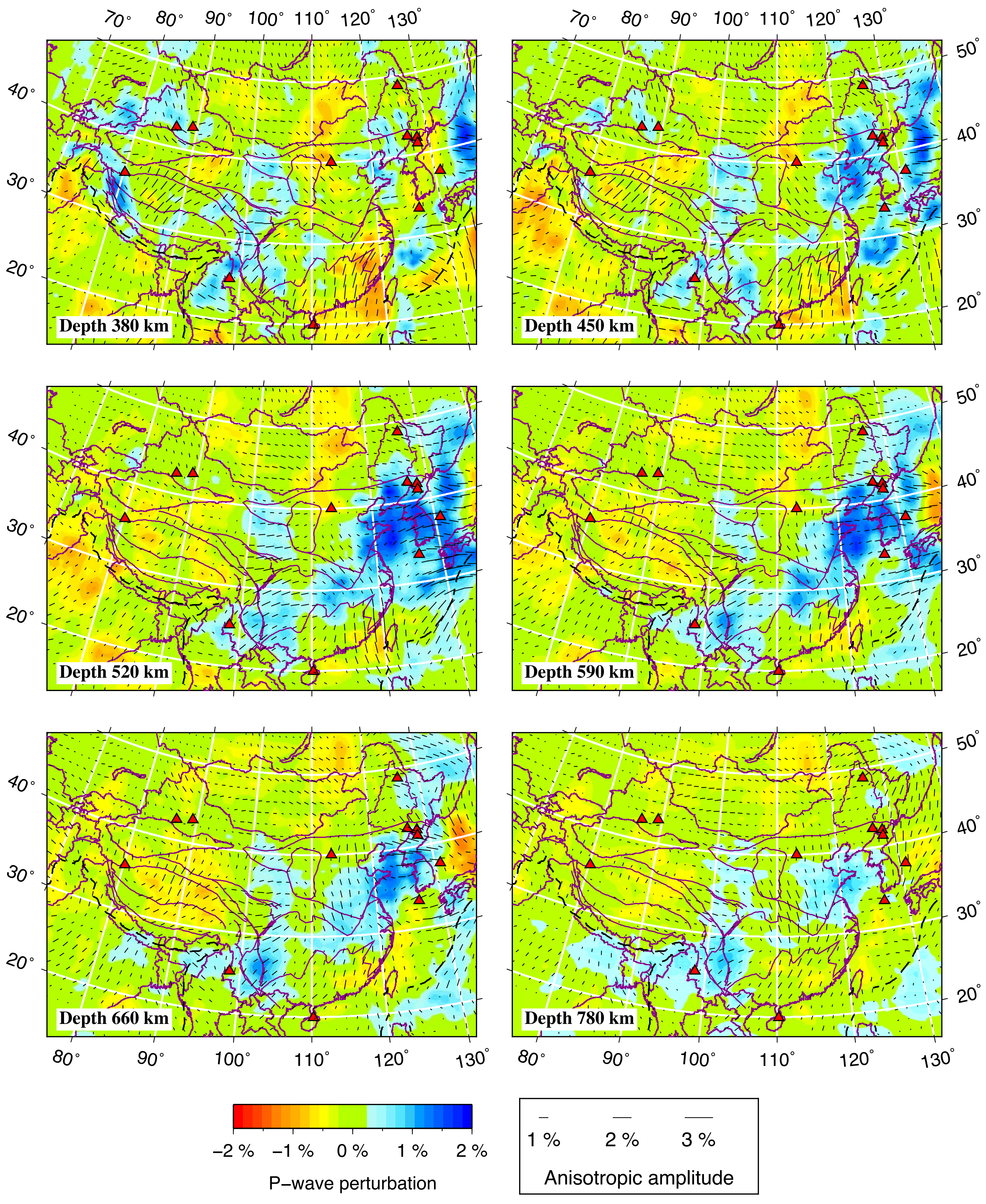

Figure S7 (continued)
